# General isochronous rhythm in echolocation calls and social vocalizations of the bat *Saccopteryx bilineata*

**DOI:** 10.1098/rsos.181076

**Published:** 2019-01-02

**Authors:** Lara S. Burchardt, Philipp Norton, Oliver Behr, Constance Scharff, Mirjam Knörnschild

**Affiliations:** 1 Institute of Animal Behavior, Freie Universität Berlin, Takustr. 6, 14195 Berlin, Germany; 2 University of Erlangen-Nuremberg, Paul-Gordan-Str. 3/5, 91052 Erlangen, Germany; 3 Smithsonian Tropical Research Institute, Barro Colorado Island, Roosevelt Avenue, Tupper Building – 401, Balboa, Ancón, Panamá; 4 Museum für Naturkunde – Leibniz Institute for Evolution and Biodiversity Science, Invalidenstraße 43, 10115 Berlin, Germany

**Keywords:** rhythm, isochronous rhythm, biomusicology, acoustic communication, echolocation

## Abstract

Rhythm is an essential component of human speech and music but very little is known about its evolutionary origin and its distribution in animal vocalizations. We found a regular rhythm in three multisyllabic vocalization types (echolocation call sequences, male territorial songs and pup isolation calls) of the neotropical bat *Saccopteryx bilineata*. The intervals between element onsets were used to fit the rhythm for each individual. For echolocation call sequences, we expected rhythm frequencies around 6–24 Hz, corresponding to the wingbeat in *S. bilineata* which is strongly coupled to echolocation calls during flight. Surprisingly, we found rhythm frequencies between 6 and 24 Hz not only for echolocation sequences but also for social vocalizations, e.g. male territorial songs and pup isolation calls, which were emitted while bats were stationary. Fourier analysis of element onsets confirmed an isochronous rhythm across individuals and vocalization types. We speculate that attentional tuning to the rhythms of echolocation calls on the receivers' side might make the production of equally steady rhythmic social vocalizations beneficial.

## 1. Introduction

Music is widespread in all human cultures but its evolutionary origin is poorly understood [1]. The field of biomusicology attempts to answer questions on the origin and purpose of music by focusing on the physiological, psychological, behavioural and evolutionary aspects of music in a comparative approach. That approach includes not only human music but musicality as a term for different traits that occur spontaneously and are based on and constrained by biology and cognition in animal vocalizations [2,3]. Music contains several key components—that can be separately investigated as musicality traits—such as pitch (governing melody and harmony), rhythm (defining temporal structure) and sonic qualities named timbre [1]. Our study focuses on rhythm as a musicality trait, probably with multiple evolutionary backgrounds [4].

Rhythm can be defined as a ‘systematic patterning of sound in terms of timing, accent, and grouping’ [5]. Overall, our intuitive understanding of rhythm concerns periodicity, which is the expectation of a recurrent event. One special kind of periodic rhythm is an isochronous beat, as produced e.g. by a metronome. In an isochronous beat, all beats have the same length and all beat-to-beat intervals have the same length [5]. When it comes to analysing animal vocalizations for rhythmicity, two questions need to be answered. (a) How well can an animal produce a certain rhythm and (b) are rhythmic patterns similar or different between vocalization types and between individuals? Another interesting comparison not regarded in this project would be between species. Furthermore, the relevance and biological constraints shaping an existing rhythm need to be discussed.

In a recent study on rhythm in song of zebra finches (*Taeniopygia guttata*), both questions were answered. Individual males had a distinct isochronous rhythm which fitted syllable onsets better than expected by chance. Distinct rhythms between individual males ranged from 10 to 60 Hz [6]. Other examples of animals producing rhythmic signals include the palm cockatoo (*Probosciger aterrimus*) which uses tools to ‘drum’ a quasi-isochronous beat on branches in a consistent context (the rhythm frequencies were not analysed in detail) [7] or chimpanzees cracking baobab fruits in a fashion probably eligible to generate individual signatures, which might help to recognize unseen companions [8]. A subsequent question would be whether animals can distinguish between rhythms, isochronous or otherwise. Rats, for example, are able to discriminate between different isochronous rhythms in a habituation–dishabituation experiment [9], while European starlings are able to discriminate between rhythmic and arrhythmic patterns [10]. Moreover, the first instance for a biologically relevant rhythm in non-human mammalian vocalizations was found in the northern elephant seal, where males can discriminate between familiar and unfamiliar male opponents using the temporal structure of vocalizations. Rhythms apparently differ between individuals in a way that facilitates discrimination of individuals [11]. Nevertheless, compared to other aspects of vocal communication, studies on rhythmicality in animals are still sparse.

Our study aims to broaden the knowledge of rhythm in animal vocalizations by investigating whether isochronous rhythms can be found in different vocalization types of bats. Specifically, we investigated how well different vocalizations of bats fit an isochronous beat and whether the patterns are similar between individuals or vocalization types.

We studied the Neotropical greater sac-winged bat *Saccopteryx bilineata* which has a rich vocal repertoire [12] and is capable of vocal production learning [13]. The species' vocal repertoire consists of distinct vocalization types that are uttered in different behavioural contexts. In this study, we focused on echolocation call sequences, isolation calls and territorial songs, all of which are multisyllabic vocalizations with clear syllable onsets. Isolation calls are produced by pups to solicit maternal care and by adult males to appease dominant conspecifics [14–16]. With a length of up to 2 s and a multisyllabic structure, isolation calls of *S. bilineata* are among the most acoustically complex bat isolation calls described [14,15]. Territorial songs are produced by adult males to repel rivals and attract mating partners [12,17]. They are acquired by imitating conspecifics’ song during ontogeny [13,17,18]. Echolocation calls are produced by male and female *S. bilineata* for orientation, navigation and insect prey capture [19]; in addition to their primary function, echolocation calls facilitate social communication among group members [20]. We chose those three vocalization types to get insight into rhythmicity in both innate vocalizations (isolation calls, echolocation call sequences) and learned ones (territorial songs) as well as to investigate potential age differences in rhythmicity (in pup isolation calls).

The individual rhythms found in zebra finch song were discussed to be potentially advantageous for anticipating events, i.e. song syllables. Tuning attention to rhythmic production could reduce ‘attentional energy’ (*sensu*: [21]) and increase signal perception [6]. Correspondingly, rhythmicity in bat vocalizations might be adaptive for saving metabolic energy because the flight is energetically costly. In many bat species, echolocation calls are coupled to wingbeat and respiratory cycle (e.g. [22–25]), which is thought to be energy efficient. Moreover, not only behavioural correlates can be found but also neuronal correlates: wingbeat and echolocation calls in *Roussettus aegyptiacus* are tightly coupled around theta frequencies (5–12 Hz, [26]), brain wave frequencies which are known to play a role in active movement and stimulus intake [27]. Consistently, preliminary data on *S. bilineata* suggests a wingbeat of around 6–12 Hz (HU Schnitzler 2018, personal communication). During search flight, one or two echolocation calls might be uttered per wingbeat, which corresponds to echolocation call intervals of 6–24 Hz (wingbeat frequencies of around 6–12 Hz) found in other studies on *S. bilineata* [19,28,29].

Because of the coupling of wingbeat and echolocation pulses, we predicted periodic, isochronous pulses (following [30]) with frequencies between approximately 6–24 Hz in echolocation call sequences of *S. bilineata*. We assumed that echolocation call sequences would fit a specific isochronous rhythm significantly better than random vocal sequences would. Moreover, we expected this rhythm to be similar between individuals due to common physiological constraints. Since social vocalizations (pup isolation calls and male territorial songs) are uttered by perched bats in the day roost, not coupled to wingbeat, we predicted to find individually different rhythms that might support vocal discrimination of different individuals, as previous research suggests.

## 2. Material and methods

### 2.1. Labelling of vocalization types

**Figure 1.**
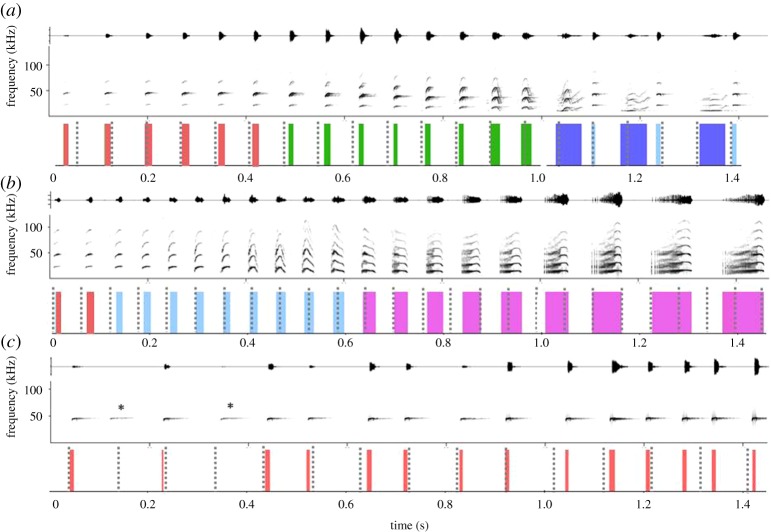
Rhythm^S^ fits well on three vocalization types: oscillograms (top rows in *a–c*) and spectrograms (middle rows) of vocalizations ((*a*): isolation call, (*b*): territorial song, (*c*): echolocation call sequence) with fitted rhythm^S^ as dotted lines in the bottom row. Element durations are indicated by coloured bars, measured from the oscillograms. Note that echoes visible in the spectrograms may make the elements appear longer than they are in the oscillograms. Different colours indicate different element types (described in earlier studies [15,31]). (*a*) Introductory elements, simple variable elements followed by composite elements and simple stereotyped elements in an alternating order. (*b*) Echolocation-like calls (comparable to the introductory elements in (*a*), short tonal elements and buzz elements. (*c*) Echolocation calls. (*) indicates two elements not being labelled due to a low amplitude.

We analysed three different vocalization types of *S. bilineata*, namely isolation calls, territorial songs and echolocation call sequences (figure 1). Isolation calls and territorial songs are multi-component vocalization types containing four different element types each, while echolocation call sequences are series of one element type with alternating frequencies.

For each vocalization, the on- and offset of its elements and the duration of the silent gaps between elements was determined for subsequent analyses. For isolation calls and territorial songs, the element on- and offsets were determined manually based on oscillograms (see [14] and [31] for details). For echolocation call sequences, we used an automatized procedure in Avisoft SASLab Pro (based on amplitude detection threshold; −20 dB relative to the call's peak frequency) to determine element on- and offsets.

We analysed isolation calls from 25 pups (10 males, 13 females, 2 not sexed) belonging to a population of wild *S. bilineata* in Costa Rica (see [14] for details on study site and sound recordings). Each isolation call contained 5–26 elements (14 ± 3.5, mean ± s.d.) and was composed of 2–4 different element types (mean: 3 element types). We followed the nomenclature introduced in an earlier study [15] and labelled the element types (a–d) as introductory elements (a), simple variable elements (b), composite elements (c) and simple stereotyped elements (d). Data for each pup consisted of 20 isolation calls, recorded at two ontogenetic stages (non-volant and volant; 10 isolation calls each). Only one call per pup and day was selected to minimize temporal dependence among vocalizations.

We analysed territorial songs of 14 adult males belonging to a population of wild *S. bilineata* in Costa Rica (see [31] for details on study site and sound recordings). Data for each male consisted of 10–11 songs, which were recorded on different days. Each song contained 6–46 elements (20 ± 8.0 mean ± s.d.) and was composed of five different element types (1–5) (mean: 3 different element types). We followed the nomenclature introduced in an earlier study [31] and labelled the element types (a–e) as short tonal elements (a), buzz elements (b), trills (c), noise bursts (d) and echolocation-like calls (e) (figure 1).

Sequences of echolocation calls were recorded from 33 wild Costa Rican *S. bilineata* (15 males, 18 females) when they were released after capture, i.e. in a non-foraging context. Calls of known individuals were recorded in standardized release situations in relatively open space (e.g. at a forest clearing). Recorded calls resembled normal search calls (see [20] for details on study sites and sound recordings). Echolocation call sequences consisted of 11–38 elements (21 ± 6.95, mean ± s.d.) with no further differentiation into different elements types. One echolocation call sequence per bat was used for further analysis.

### 2.2. Assessment of best-fitting rhythms

**Figure 2.**
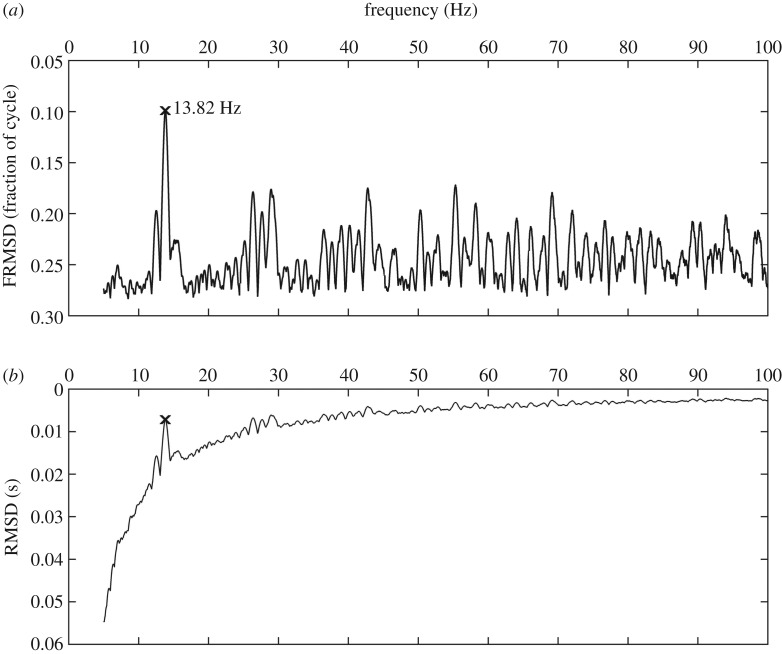
Optimization process: best-fitting rhythms were found by selecting the rhythm with the lowest corresponding FRMSD (black cross with corresponding rhythm^S^), the frequency-normalized root-mean-square deviation (*a*); (*b*) shows the corresponding RMSD values.

Simply analysing inter-onset intervals of social vocalizations, as is often done for echolocation call sequences (e.g. [19,28,29]), is problematic because this would oversimplify the temporal structure of multisyllabic social vocalizations with strongly varying syllable durations. Other approaches to analyse the temporal structure of animal vocalizations include generate-and-test approaches or Fourier analysis [32]. We chose a generate-and-test approach (GAT approach) originally developed for rhythm analysis in zebra finch song [6]. The GAT approach allowed us to find an isochronous rhythm (i.e. a pattern with equal time intervals) that best fitted the onsets of elements in a given sequence. We named this best-fitting rhythm ‘signal-derived rhythm’ or rhythm^S^ (same as pulse^S^ in [6]). The GAT approach was performed by a custom Matlab program (see [6] §3.7 for more details). It creates isochronous pulse trains in 0.01 Hz increments in a predefined frequency window of 5–100 Hz (i.e. 5–100 pulses per second). The lower range of rhythm frequencies was determined by expected values [19,28,29] the upper range experimentally by testing different ranges. 100 Hz was deemed appropriate because, when testing for up to 200 Hz only very few values for best-fitting rhythms lay above 100 Hz. Restricting the frequency window was a question of minimizing computing time. For each rhythm, temporal deviations of each element to the nearest pulse gave an overall root-mean-square deviation (RMSD). Pulses were offset (+ one phase in 1 ms steps, see [6]) to minimize the RMSD. As RMSD is negatively correlated with rhythm frequency (i.e. faster rhythms generally result in lower RMSD values; figure 2*b*), we normalized the RMSD by multiplying it by the respective rhythm frequency, yielding a measure for deviation relative to the rhythm period; it describes the average temporal deviation as a fraction of a full cycle. The resulting frequency-normalized RMSD (FRMSD) was used to assess the goodness-of-fit for each rhythm: the lowest FRMSD indicated the best-fitting rhythm frequency. This way the slowest isochronous rhythm, coinciding best with element onsets, was found (figure 2).

### 2.3. Clustering

A visual examination of the resulting best-fitting rhythm^S^ indicated an accumulation of certain frequency values for each individual and vocalization type. Rhythm frequencies showed a strong right skewness, which is why common measures such as mean, or median would have been inaccurate. Therefore, we performed a cluster analysis to assess whether specific rhythm frequency clusters existed. We applied an agglomerative, hierarchical clustering algorithm which used the group average of frequency distances as a dissimilarity measure (dissimilarity threshold was set to 0.05 for all datasets). The frequency data were log10-transformed before clustering because an earlier study [6] showed that log10-transformation resulted in comparable clusters for different frequencies because these clusters had the least frequency-dependent standard deviation.

### 2.4. Modelling

To confirm that the rhythm frequencies obtained by the GAT approach are an inherent property of the respective vocalization type and cannot be found in arbitrary element sequences, we created artificial temporal vocalization patterns based on the previously measured element and gap durations, assessed their FRMSD values and compared them to the FRMSD values of the original vocalization types. We created two different types of artificial vocalization patterns that were used in different models: in Model 1, we used artificial vocalization patterns with the randomized element and gap duration but intact sequence information (i.e. the correct order of consecutive elements); in Model 2, we used artificial vocalization patterns where each element and gap were replaced with a random duration, irrespective of element type and sequence. Model 2 did not apply to echolocation call sequences because they consisted of only one element type repeated in series, thus making the dismissal of sequence information pointless. Element and gap durations for both models were drawn randomly out of the pool of original recorded durations of the same type from all individuals (elements a–e and gaps following elements a–e, respectively). The respective pool from which durations were drawn contained only element and gap durations of the vocalization type (isolation calls, territorial songs or echolocation call sequences) to be modelled.

For each vocalization, we ran both Model 1 and 2 (not for echolocation call sequences, see above) 50 times. For every iteration, a new FRMSD value was obtained. We calculated the means of all model FRMSD values per individual and compared them to the means of all original FRMSD values per individual.

### 2.5. Fourier analysis

Results of the GAT approach were compared to FFT analyses of all sequences (following [6,33]). Timestamps of element onsets were used to form a binary point process. We created strings with a time resolution of 5 ms in which only events (i.e. element onsets) were represented by ‘1’, everything else in the string was represented by ‘0’. The higher the temporal resolution of the input data, the lower the frequency resolution of the FFT output will be. With the sequence lengths available to us, a time resolution of 5 ms proved to be the best compromise between the two constraints. After calculating a fast Fourier analysis, frequencies of maximum power were selected as best-fitting rhythms and the pattern compared to GAT-results. A customized Matlab script was used for the analysis.

### 2.6. Statistics

Data distribution was assessed using a Shapiro–Wilks test for all datasets. Artificial data from both randomizations (1 and 2) were compared to original data with repeated measures ANOVA (Tukey's *post hoc* comparison) for isolation calls and territorial songs. Echolocation call sequences were tested against randomization 1 via a Welch-corrected *t*-test because variances differed significantly. A paired *t*-test was used to compare the results of different ontogeny stages in isolation calls. Statistical differences were considered significant for *p* < 0.05 (*p < 0.05, **p < 0.01, ***p < 0.001). When random numbers were needed, those were generated using the R-function ‘runif’.

### 2.7. Software

For analyses and preparing figures, we used Matlab (Version 2016b & 2015b), R (v. 3.5.1), GraphPad v. 5 and Avisoft SASLab Pro v. 5.2.10. Customized Matlab programs written by Philipp Norton (PN) and Lara Sophie Burchardt (LSB) were adjusted and used for the rhythm optimization (PN), model calculations (PN & LSB), FFT analysis (LSB) and cluster visualization (PN).

## 3. Results

### 3.1. Isochronous rhythm

**Figure 3.**
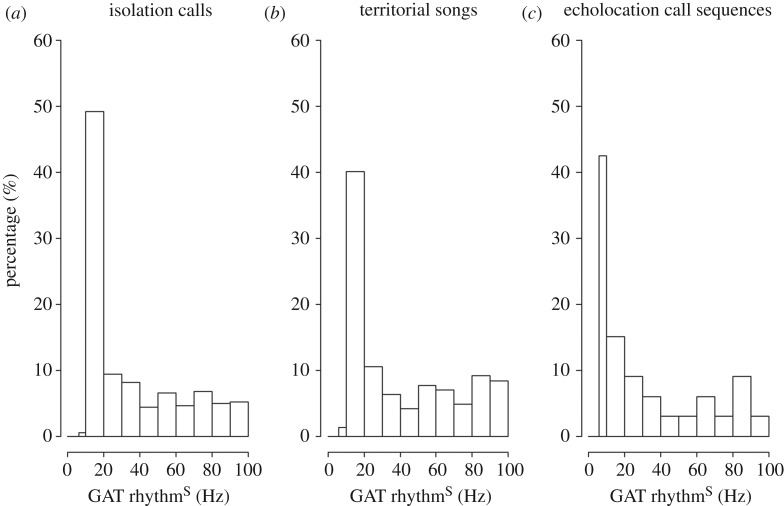
GAT analysis: regular rhythms in *S. bilineata* vocalizations. The relative majority of calls/songs occurred in rhythm frequencies below 20 Hz for all vocalization types.

**Figure 4.**
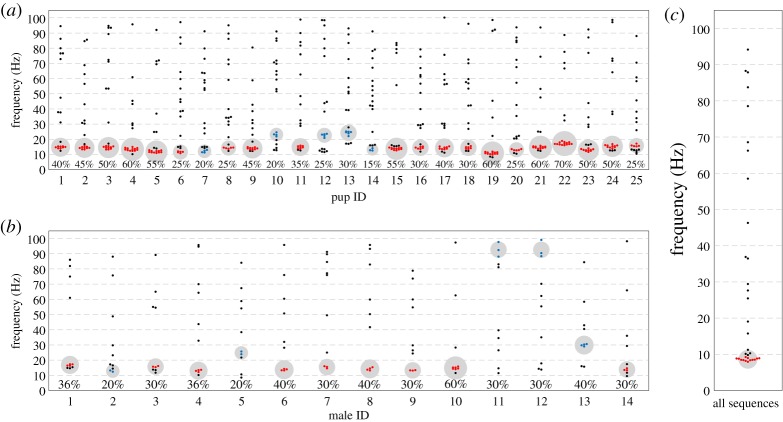
Isochronous beat in bat vocalization: (*a*) rhythm clusters in isolation calls of *S. bilineata* pups; (*b*) rhythm clusters in territorial songs of *S. bilineata* males; (*c*) rhythm clusters in echolocation call sequences of *S. bilineata* adults. Marked in red are the data belonging to the largest cluster containing at least 25% of songs/calls, within the range of 6–20 Hz. Marked in blue are the data belonging to the largest cluster that were not considered. The percentage of data in the largest cluster is shown at the bottom of each column. The area of circles is scaled to the percentage of calls/songs in the respective clusters.

For each vocalization, we found an isochronous rhythm (rhythm^S^) that coincided best with the onsets of elements (electronic supplementary material, audio files A1–3). A rhythm^S^ between 6 and 20 Hz dominated across individuals as well as across vocalization types: 49.4% of isolation calls (247 out of 500 calls), 41% of territorial songs (59 out of 143 songs) and 57% of echolocation call sequences (19 out of 33 sequences) had a best-fitting rhythm of 6–20 Hz (figure 3). Corresponding results were obtained when focusing on individuals instead of vocalization types. Twenty out of 25 pups produced isolation calls which clustered predominantly in the frequency range of 10–20 Hz; the largest clusters contained 25–70% of calls per pup (figure 4*a*). Nine out of 14 males produced territorial songs which clustered predominantly in the frequency range of 10–20 Hz; the largest clusters contained 30–60% of songs (figure 4*b*). We considered clusters with their mean falling into the range between 10 and 20 Hz and the cluster comprising at least 25% of data (clusters are marked in red in figure 4*a–c*). Echolocation call sequences clustered predominantly in the frequency range of 6–20 Hz, 39% of sequences making up the strongest cluster (between 6 and 10 Hz), adding up to 57% between 6 and 20 Hz. Note that echolocation call sequences were pooled over all individuals (figure 4*c*, see Material and methods).

### 3.2. Comparison of artificial randomized vocalizations

To confirm that the observed element onsets in *S. bilineata* vocalizations aligned to an isochronous rhythm well and more closely than expected by chance, we compared the FRMSD values of artificial vocalization types with the FRMSD values of the original vocalization types. All artificial vocalization types had randomized element and gap durations; sequence information, i.e. the consecutive order of elements was either preserved (Model 1) or ignored (Model 2).

**Figure 5.**
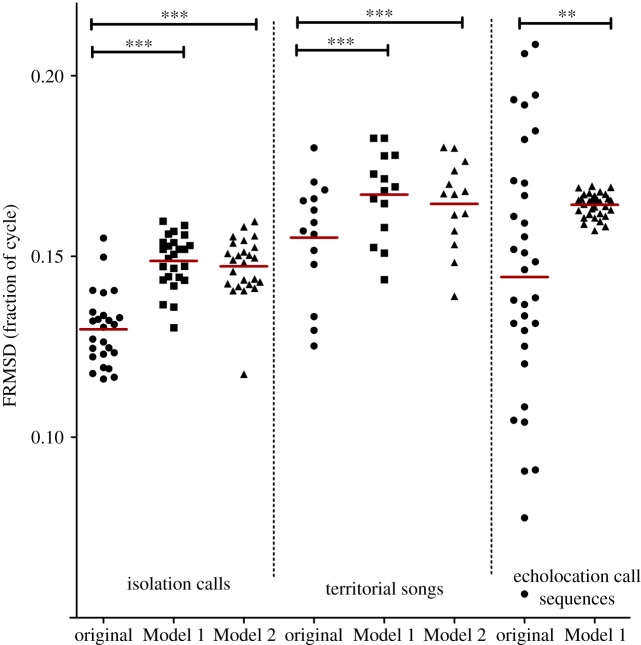
Model validation: mean values for FRMSD, comparing original data to ‘bat-like’ artificial data (Model 1: intact sequence information, Model 2: random sequences). Original data showed significantly lower deviations (*p < 0.05; **p < 0.01; ***p < 0.001). Depicted are means per individual for isolation calls and territorial song, and best-fitting rhythms of single sequences for echolocation call sequences, explaining the higher spread. Red lines indicate the respective mean of a dataset.

As expected, original vocalizations had significantly lower FRMSD values than artificial Model 1 or Model 2 vocalizations (repeated measures ANOVA: isolation calls: *F* = 71.17, d.f. = 74, *p* < 0.0001; territorial songs: *F* = 30.38, d.f. = 41, *p* < 0.0001; unpaired *t*-test (with Welch correction): echolocation call sequences: *t* = 2.35, d.f. = 33, *p* = 0.0023), indicating that the element onsets of original vocalizations matched an isochronous rhythm more closely than expected by chance (figure 5).

### 3.3. Fourier analysis

Results of the fast Fourier analysis of a binary point process string where element onsets were represented by ‘1’ resulted in the same if not stronger picture at the level of vocalization types. 55.4% of isolation calls, 47.8% of territorial songs and 66% of echolocation call sequences showed a dominant rhythm between 6 and 20 Hz (54% between 6 and 10 Hz) (electronic supplementary material, figure S1).

### 3.4. Ontogeny effect

Furthermore, we ran statistical analyses to investigate the effect of ontogeny on rhythmicity for pup isolation calls. For each pup, we compared the frequencies of isochronous rhythms of the first and last two isolation calls recorded during ontogeny (non-volant phase and volant phase). Rhythm^S^ frequencies in isolation calls did not change significantly during the pups' ontogeny (paired *t*-test: *t* = 1.31, d.f. = 49 *p* = 0.20, electronic supplementary material, figure S2).

## 4. Discussion

The novel aspect presented in this study is the documentation of isochronous rhythm patterns in different vocalization types of the bat *S. bilineata*. With a GAT approach as well as an FFT analysis, vocalizations were analysed to find a best-fitting rhythm over a wide frequency range of 5–100 Hz (i.e. pulses per second). Even though the three analysed vocalization types (pup isolation calls, male territorial songs and echolocation call sequences) differed in their acoustic structure and the behavioural situation they were produced in, their best-fitting rhythms fell in a quite narrow frequency window. Element onsets coincided best with rhythm frequencies between 6 and 20 Hz, independent of vocalization type and vocalizing individual. Analyses showed that rhythm frequencies were most abundant between 6 and 10 Hz for echolocation call sequences and between 10 and 20 Hz for territorial songs and isolation calls. The same picture was found with an FFT analysis at the level of vocalization types.

Therefore, the best-fitting rhythms were comparatively similar across vocalization types and vocalizing individuals in *S. bilineata*, with social communication signals showing rhythms twofold of echolocation call sequences. Other studies on rhythmicality in animal vocalizations so far did show patterns that differed between individual animals [6], and temporal structure, namely the rhythm, may be used by conspecifics for individual discrimination [11]. A biological constraint shaping rhythms to be more alike between individuals is not apparent. Since there are not many comparable studies yet, our results might prove to be the rule rather than an exception.

Nevertheless, the pattern of rhythm^S^ in the analysed vocalizations could be caused by physiological constraints and/or mechanisms to save energy. The production of echolocation calls when a bat is searching for prey items but has not detected anything yet is correlated with respiration which, in turn, is tightly coupled to wing beat. For many bat species, a 1:1 relation has been found (e.g. [23]). The soprano pipistrelle (*Pipistrellus pygmaeus*), for example, produces one or two echolocation calls per wingbeat and respiratory cycle [25]. In other pipistrelle bats (*P. pipistrellus, P. kuhlii, P. nathusii* [24]), greater horseshoe bats (*Rhinolophus ferrumequinum*), little brown bats (*Myotis lucifugus*), Parnell's mustached bats (*Pteronotus parnellii rubiginosus*) and Seba's short-tailed bats (*Carollia perspicillata* [22]) wingbeat and echolocation calls are also coupled. Coupling was also found in the tongue-clicking pteropodid bat *Rousettus aegyptiacus,* indicating that a strong coupling of wing beat, respiration and sonar emission is widespread in bats regardless of sound production mechanism.

In *S. bilineata,* respiratory cycle and wing beat are between 6 and 12 Hz during search flight (HU Schnitzler 2018, personal communication). Our results suggest that in the release situation in which the echolocation call sequences were recorded, bats mainly uttered one call per wingbeat, which fits the low sensory needs in the relatively open space in which releases took place. In a situation with higher sensory needs, expected rhythm frequencies should be doubled, i.e. lie between 12 and 24 Hz, most of which overlaps strongly with the rhythm frequencies found in the social vocalizations. Therefore, we argue that the rhythm frequencies most abundant in social vocalizations (10–20 Hz) and in echolocation call sequences during search flight (6–10 Hz and, to a lesser degree, 10–20 Hz) can be regarded as comparatively similar.

During prey capture, however, echolocation call sequences contain not only search flight calls but also approach flight calls (when prey has been detected and is approached) and a so-called final buzz (immediately before prey capture, very short and broadband echolocation calls with extremely short IOIs are produced), which enhances the sensory information available for the foraging bat. Even though wing beat, respiratory cycle and sonar emission are tightly coupled during search flights (probably to increase energy efficiency), this might not provide sufficient sensory information during prey capture, i.e. in a situation where high temporal resolution is needed (per wing beat and respiratory cycle up to 10–15 pulses can be emitted [24]). A larger ratio between wing beat, respiratory frequency and emitted echolocation calls could result in a weaker rhythmic pattern in our analyses. In the approach phase, the number of echolocation calls per wing beat can vary widely, depending on the current sensory needs of a foraging bat. Therefore, it seems reasonable to assume that echolocation call sequences during prey capture do not follow any clear rhythm but strongly depend on the bats’ current sensory needs. This could easily be tested on echolocation call sequences recorded in foraging situations. Correspondingly, a previous study on the big brown bat *Eptesicus fuscus* showed that the strict 1 : 1 synchronization of wing beat, respiration and call emission was not found during complex navigation tasks, where freely behaving individuals had to search for prey (tethered mealworms, suspended at about 1.5 m height) in a flight room, equipped with various obstacles, such as artificial houseplants [34]. During search flights, however, metabolic needs, e.g. being energy efficient, may play a more important role [35]. To investigate the task/situation dependence of the coupling of wing beat, respiration and call emission, it would be worthwhile to analyse rhythm^S^ of echolocation call sequences produced in a feeding context in bat species in which a strict 1 : 1 coupling has been found during search flight.

The determination of rhythm^S^ (method developed by [6]) could be a valuable addition to currently used methods because it is not dependent on a laboratory setting. Knowledge of wing beat and/or respiratory rates could be combined with analyses of rhythm^S^ of echolocation call sequences and social vocalizations recorded from freely behaving, wild bats to gain insights on coupling relations in natural situations. Especially for more complex vocalization types with the variable element and gap durations, the GAT approach and FFT analysis provide a more detailed picture than simply assessing IOIs. The latter method ignores the sequential structure of vocalizations and their variable element durations, potentially concealing higher order regularity.

To assess the goodness-of-fit for our analyses of rhythm^S^, we compared deviations from rhythm^S^ of original and artificially created vocalizations that were randomly drawn from a pool of typical element and gap durations. Original vocalizations deviated significantly less from rhythm^S^ than did artificial vocalizations (i.e. element onsets of original vocalizations coincided significantly better with an isochronous rhythm than artificial vocalizations), indicating that the rhythm^S^ found in *S. bilineata* vocalizations was not an artefact of the typical duration and sequence of this species' vocalizations.

One aspect worthy of discussion is the relation between rhythm frequencies of echolocation call sequences produced by *S. bilineata* during search flight (which were coupled to wing beat frequencies) and social vocalizations produced by individuals hanging in their day roost (pup isolation calls and male territorial songs). We doubt that rhythm frequencies of isolation calls and territorial songs are caused by a coupling of sound emission to respiration because echolocation calls produced by roosting bats can occur at any point in the respiratory cycle [23]. Taking this into account, it seems reasonable to assume that social calls can be emitted at any point in the respiratory cycle as well. Nevertheless, as stated before, we argue there is a relation between the dominant frequencies of the three vocalization types, and we regard them as being comparatively similar. The similarity of rhythm frequencies could suggest a common evolutionary background, which might be the coupling between respiration, wingbeat and echolocation call emission. However, increasing evidence suggests that flight preceded echolocation [36,37], which would indicate that vocal communication preceded echolocation as well (assuming that bats’ predecessors communicated with social calls, as many small mammals do). It is therefore possible that social calls, despite being probably phylogenetically older than echolocation, adopted the rhythm frequencies of echolocation calls at some point.

It is interesting to compare the strength of rhythms between isolation calls and territorial songs because isolation calls are produced within minutes after birth [15] while territorial songs are learned during ontogeny [13]. Generally, a higher variability in rhythm^S^ may be expected when comparing learned vocalizations to innate vocalizations. In our study, rhythm frequencies predominantly clustered between 6 and 20 Hz, but cluster strength of individuals was on average lower in territorial song than in isolation calls (37% in territorial song compared to 44.7% in isolation calls; GAT approach). This difference in individual cluster strength resembled the overall difference between both vocalization types, because only 41.6% of all territorial songs had rhythm frequencies between 6 and 20 Hz, while 49.8% of isolation calls did.

Rhythmic properties of echolocation could represent the same neuronal correlates underlying production of social vocalizations. In the Egyptian fruit bat (*R. aegyptiacus)* wingbeat and tongue clicks are tightly coupled around 10 Hz [26], as we found for *S. bilineata*. These rhythm frequencies show a resemblance to the frequency of theta brain waves. Thought to be important for movements, spatial memory and active stimulus intake [27] among others, theta waves might be a promising neural correlate explaining the production of the detected rhythms.

It might be advantageous to produce rhythmic vocalizations because ‘rhythmic attention’ (*sensu* [38]) helps receivers to decode rhythmic signals easier and faster [39]. The attention of receivers cycles in an oscillatory way when a rhythm exists (e.g. [40,41]). Since rhythmic signals are predictable, ‘rhythmic attention’ enables receivers to provide most ‘attentional energy’ at a time point where the next stimulus is to be expected. This is advantageous because cognitive capacities are limited [42] and an optimization of attention timing is helpful to not miss relevant stimuli. For example, when humans were asked to assess the difference in pitch of two focal tones separated by regularly timed tones, the assessment of pitch difference was better when the second focal tone followed the regular timing of the separating tones than when was slightly displaced from the regular timing [43]. Another example from macaques shows that neuronal oscillations in the primary visual cortex entrain to a stimuli stream (visual stimuli) when the stream is rhythmic, a mechanism resulting in decreased reaction time and an increase in the response gain for events that are task relevant [44]. Bats' attention as well as the auditory system collectively could be tuned to echolocation rhythms, because bats are exposed to those rhythms for large parts of their lives [45]. Therefore, it might be advantageous to produce vocalizations in the same frequency window to increase detection by receivers. At the moment, we do not know whether rhythmic attention plays a role in *S. bilineata*. Playback experiments violating expected rhythmic patterns in social vocalizations or direct assessment of the animals’ rhythm perception would be a valuable avenue for future research. A switch from a rhythm determined by physiological constraints to a rhythm decoupled from its original production constraints but still with an adaptive function (e.g. rhythmic attention) might have been one step during evolution that paved the way to develop music as we know it.

In summary, this study demonstrates an isochronous rhythm in three bat vocalization types in which metabolic constraints leading to rhythmic patterns are more (echolocation calls) or less (isolation calls, territorial songs) likely. The two methods used in this study (GAT and FFT) enable the analysis of best-fitting rhythms in a corresponding way. Future studies should profit by complementary use of both methods in addition to IOI assessment. To further study the coupling or decoupling of wing beat, respiration and sound emission in animals as well as its biological relevance, it would be highly beneficial to compare different species of bats and birds which sing in flight as well as other echolocating mammals. Such a comparative approach could provide valuable insights into the origin and relevance of rhythmicality in animals [4].

## Supplementary Material

Audio File A1: Isolation Call with overlayed optimal pulse

## Supplementary Material

Audio File A1: Territorial song with overlayed optimal pulse

## Supplementary Material

Audio File A1: Echolocation call sequence with overlayed optimal pulse

## Supplementary Material

Supplementary Materials and Figures

## Supplementary Material

Dataset

## Data accessibility

The dataset supporting this article has been uploaded as part of the electronic supplementary material. The code (GAT approach) with detailed explanations was already published in a previous publication [32].
