# Supplementary material for: General isochronous rhythm in echolocation calls and social vocalizations of the bat Saccopteryx bilineata
Source: R Soc Open Sci. 2019 Jan 2;6(1):181076. doi: 10.1098/rsos.181076 (PMC6366212; doi:10.1098/rsos.181076)
Supplement: Supplementary Materials and Figures [file rsos181076supp4.docx]

**SUPPLEMENTARY INFORMATION**

**Royal Society Open Science**

**General isochronous rhythm in echolocation calls and social vocalizations of the bat *Saccopteryx bilineata***

Lara S. Burchardt*^1^, Philipp Norton^1^, Oliver Behr^2^, Constance Scharff^1+^, Mirjam Knörnschild ^1, 3, 4+^

^*^ Corresponding author: l.s.burchardt@gmx.de

^+^ Joint senior authors.

^1^ Institute of Animal Behavior, Freie Universität Berlin, Takustr. 6, 14195 Berlin, Germany

^2^ Chair of Sensor Technology, University of Erlangen-Nuremberg, Paul-Gordan-Str. 3/5, 91052 Erlangen, Germany

^3^ Smithsonian Tropical Research Institute, Barro Colorado Island, Roosevelt Ave., Tupper Building - 401, Balboa, Ancón, Panamá.

^4^ Museum für Naturkunde - Leibniz Institute for Evolution and Biodiversity Science, Invalidenstraße 43, 10115 Berlin, Germany.

**This supplementary information contains:**

Supplementary Audio Files A1-A3

Validation Analyses – Tempo Changes

Supplementary Figures S1-S4

**Supplementary Audio Files**

**Audio File A1:** Isolation call of *S. bilineata* pup overlaid with an isochronous rhythm of 12.3Hz. The recording is slowed down to 10% of its original speed.

**Audio File A2:** Territorial song of *S. bilineata* male overlaid with an isochronous rhythm of 15Hz. The recording is slowed down to 10% of its original speed.

**Audio File A3:** Echolocation call sequence of adult *S. bilineata* overlaid with an isochronous rhythm of 8.8 Hz. The recording is slowed down to 20% of its original speed.

**Validation analysis - Tempo changes**

To assess tempo changes within vocalizations, we calculated linear regressions for the Inter-Onset-Intervals (IOI) sequences to test whether these were significantly different from zero (using an F-test), which would indicate a significant change in tempo. We conducted this analysis for a random subset of each vocalization type (two sequences per individual for isolation calls and territorial songs, all data for echolocation call sequences). To corroborate results from the tempo analysis, individual syllable deviations of the first, middle and last syllable were compared per vocalization type by means of a Friedman test; this was done to test whether deviations changed throughout a syllable sequence. This analysis was conducted on a subset of the data, chosen in the same way as for the tempo analysis.

The majority of isolation calls (74%) had a stable tempo, 22% of calls showed a decrease in tempo and 4% of calls an increase. On the contrary, the majority of territorial songs (79%) decreased in tempo, especially in the last fifth of songs (Supplementary Figure S3). However, inter-onset intervals did not increase continuously but rather abruptly, often doubling and quadrupling. These multiples of inter-onset intervals make it unlikely that the observed change in tempo had a negative effect on rhythm^S^ in our study. Furthermore results were confirmed by the FFT analysis, which is stable against tempo changes.

To corroborate that changes in tempo did not affect rhythm^S^, we calculated individual element deviations to the nearest single pulse. Element deviations did not change throughout vocalizations, since a best fitting rhythm was found by an optimization task regarding all elements of a sequence. Nevertheless, individual element deviations of vocalizations with tempo changes (territorial songs) did not differ from vocalizations without tempo changes (isolation calls) (Kruskal-Wallis, p=0.78, F=2.47, df=6, Supplementary Figure S4), suggesting that changes in tempo played a negligible role in our study. Another argument for this interpretation is the results from FFT analysis. Since the same pattern was found with a method in which tempo changes cannot affect the outcome, it is reasonable to say that tempo changes did not influence the results from GAT analysis in a crucial way.

**Supplementary Figures**

**
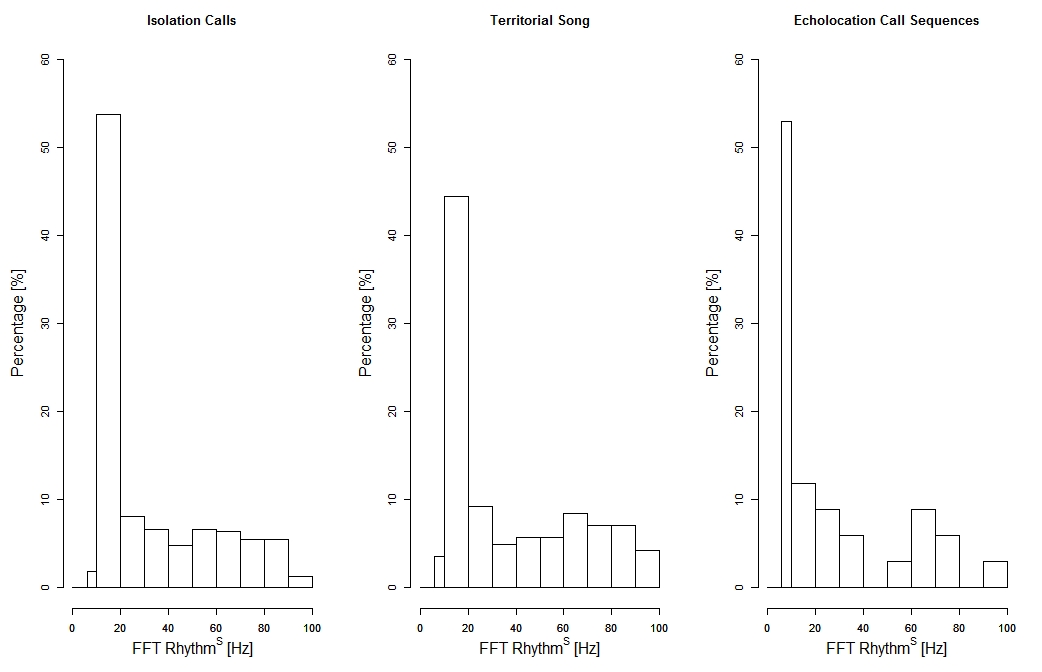
**

**
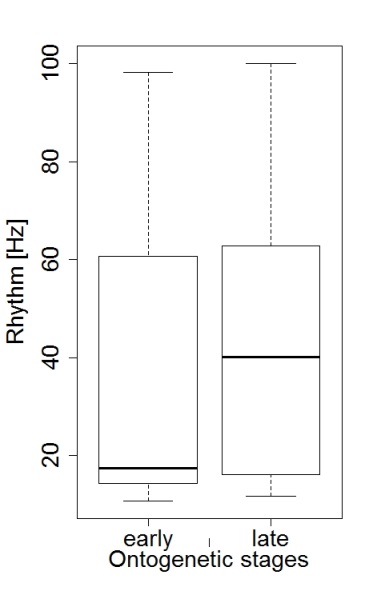
Figure S1:** FFT analysis: Regular rhythm^S^ in bat vocalizations. The relative majority of calls/songs occur in rhythm frequencies below 20 Hz for all vocalization types.

**Figure S2:** Effect of ontogenetic stage on rhythm in pup isolation calls. Early ontogeny did not differ from late ontogeny. Medians, interquartile range (25-75%) and whiskers (0-100%) are shown.

**
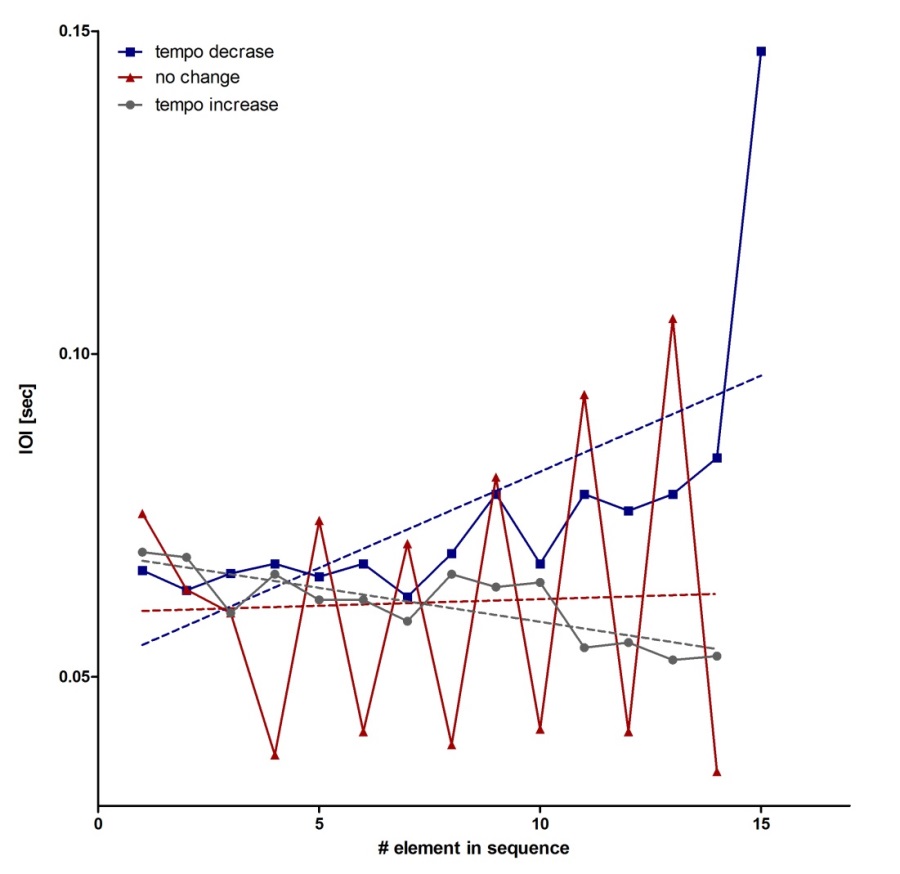
Figure S3:** Tempo changes in sequences: Three IOI sequences are shown as solid lines; dashed lines show corresponding linear regressions. Slopes of regression lines were tested against zero. Significant difference from zero was interpreted as tempo change. In red (triangle) an isolation call with no tempo change is shown, in grey (circle) an isolation call increasing in tempo and in blue (square) a territorial song decreasing in tempo rather abruptly are shown.


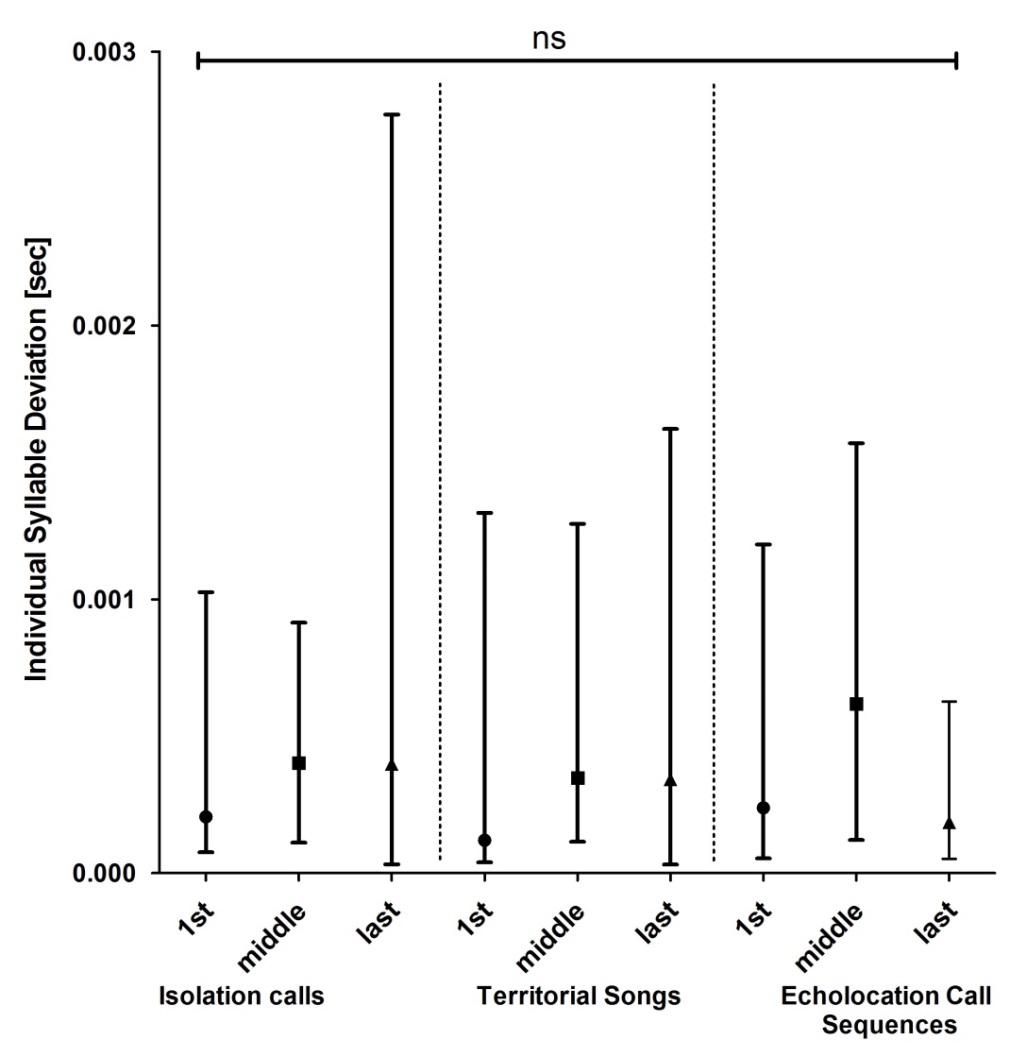


**Figure S4:** Syllable deviations of individual syllables. Individual deviations from rhythm^S^ of first, middle and last syllable of calls/songs were compared. Median and interquartile range are shown. No significant differences were found.
